# Indication of clear aligners in the early treatment of anterior crossbite: a case series

**DOI:** 10.1590/2177-6709.25.4.033-043.oar

**Published:** 2020

**Authors:** Edoardo Staderini, Romeo Patini, Simonetta Meuli, Andrea Camodeca, Federica Guglielmi, Patrizia Gallenzi

**Affiliations:** 1 Università Cattolica del Sacro Cuore, Facoltà di Medicina e Chirurgia, Istituto di Odontoiatria e Chirurgia Maxillo-Facciale (Roma, Italy).

**Keywords:** Orthodontics, interceptive, Malocclusion, Orthodontic appliances, removable

## Abstract

**Introduction:**

Anterior crossbite (AC) is defined as a reverse sagittal relationship between maxillary and mandibular incisors. According to an evidence-based orthodontic triage, the treatment need of AC is indicated if any occlusal interference is forcing the mandible towards a Class III growth pattern. Removable and fixed appliances have been suggested to correct AC.

**Objective:**

The present report aims at presenting the benefits of an alternative therapy for the early treatment of anterior crossbite using clear aligners.

**Methods:**

Two cases of anterior crossbite corrected using clear aligners in 8-years-old children are presented.

**Results:**

In both cases, AC was successfully corrected within 5 months. At the end of the treatment, overjet and overbite were corrected. No major discomfort or speech impairment was noticed by the parents.

**Conclusions:**

Due to the perceived shortcomings of alternative approaches, the use of clear aligners for correcting AC in mixed dentition should be considered as a comfortable and well tolerated appliance for young patients.

## RESUMO

**Introdução:**

A mordida cruzada anterior (MCA) é definida como uma relação sagital reversa entre os incisivos superiores e inferiores. De acordo com a evidência científica, o tratamento da MCA é indicado em casos em que a interferência oclusal favorece o crescimento mandibular em direção a um padrão de Classe III. A literatura descreve diversos aparelhos removíveis e fixos para a correção dessa má oclusão.

**Objetivo:**

A presente série de casos tem como objetivo apresentar os benefícios de uma terapia alternativa para o tratamento precoce da MCA por meio do uso de alinhadores transparentes.

**Métodos:**

Apresentação de dois casos de MCA tratados com alinhadores transparentes em crianças de 8 anos de idade.

**Resultados:**

Em ambos os casos, a MCA foi corrigida com sucesso em um período de 5 meses. Ao fim do tratamento, obteve-se sobressaliência e sobremordida ideais. Segundo o relato dos pais, nenhum grande desconforto ou comprometimento na fala foi observado.

**Conclusões:**

Tendo em vista os problemas associados a algumas abordagens alternativas, o uso de alinhadores transparentes para correção da MCA durante a dentição mista pode ser considerado confortável e bem tolerado por pacientes jovens.

## INTRODUCTION

Early orthodontic treatment in mixed dentition is indicated to reduce or even eliminate the need for further orthodontic treatment by preventing functional problems or anomalies.

Anterior crossbite (AC) is defined as a reverse sagittal relationship between maxillary and mandibular incisors. 

AC exhibits dental, skeletal, or functional aetiology or a combination of those aspects. AC of dental origin can arise by alteration of tooth inclination; skeletal AC involves a basal bone discrepancy in the sagittal plane. Functional AC (or pseudo-Class III) involves occlusion interferences that results in a mandibular displacement on closure.^1^

Orthodontists are often called upon to swiftly recognize and manage AC that may, if untreated, contribute to the development of a true Class III malocclusion and temporomandibular symptoms.^2^ Chronic trauma may affect teeth with improper tooth inclination, resulting in periodontal problems, tooth wear, an increased risk of dental fractures, bruxism, and unfavorable oral habits such as lip biting.^3^

Another benefit of early correction of AC is the possibility of alleviating posterior crossbites induced by occlusal interferences and anterior mandibular shift.^4^

Currently, clinical management of AC can be achieved with multiple treatment options. According to Wiedel et al.,^5^ ^,^ ^6^ appropriate criteria/requirements for an optimal orthodontic therapy are clinical effectiveness, long-term stability, positive cost-benefit ratio, and high patient acceptance, i.e., minimal perceived pain and discomfort.

Orthodontic fixed appliances (FA) include segmental techniques: 2 by 4 approach, with brackets bonded to the incisors and the first molars; and 2 by 6, including first molars and the 6 anterior (primary or permanent) teeth. To raise the bite, the fixed appliance treatment is frequently combined with a composite coverage temporarily bonded to the occlusal surfaces of posterior teeth.

Wiedel et al.^7^ showed that the average duration of FA treatment, including the 3-month retention period, is 5.5 months, and a small number of minor complications (bond failures) is observed. Fixed appliance is the gold-standard treatment for children with whom compliance problems with wearing removable appliances (RA) are anticipated. As an impact of oral health-related quality of life, patients reported more discomfort eating different kinds of hard and soft food; poor oral hygiene can lead to decalcification and caries.^5^

Removable appliances include acrylic plates endowed with anterior springs that deliver light-continuous tipping movements to each incisor in an anterior crossbite.^2^ The protrusion springs can be activated at each monthly visit until normal incisor overjet is achieved. The RA comprises a bilateral occlusal coverage, which allows the ‘jumping’ of the bite by increasing the occlusal vertical dimension. The RA must always be worn, except during meals and toothbrushing. If the patients’ compliance in wearing the appliance is optimal, a successful correction of AC is accomplished in approximately 6.9 months.^7^

Patient-related complications (distortion/breakage/loss of the appliance, and low wear-time adherence) can be expected from a removable appliance, and patients report difficulties talking or doing school and leisure activities.^7^

In recent years, treatment approaches have been expanded with the use of clear aligners. The aesthetics, comfort, and oral hygiene of clear aligners are superior to conventional fixed appliances.^8,9^ As it regards patient’s perception, the impact of the clear aligners’ treatment in daily activities (oral symptoms, functional limitations) is suggested to be lower than a multi-bracket treatment, especially in the first 6 months of therapy.^10^

This case series aims to present the results of early orthodontic treatment of two anterior crossbite cases performed with clear aligners.

## MATERIAL AND METHODS

The present patients were clinically assessed and fully investigated regarding oral hygiene, general health along with any associated family history of Class III presentations. In accordance with the British Orthodontic Society Radiographic Guidelines (*https://www.bos.org.uk*), lateral cephalometric radiographs were obtained and analysed. Landmarks and measurements were validated by Shaw et al,^11^ and all data were anonymized.

To diagnose any functional shift of mandible caused by AC with dental aetiology, the clinicians guided the mandible to seat the condyles into centric relation and evaluated any change in the molar and incisor relationship from centric occlusion to maximum intercuspation. This maneuver is also useful to estimate the sagittal and transversal jaw discrepancy based on clinical evaluation.

## TREATMENT ALTERNATIVES

The main concern of both patients was the unaesthetic appearance of the maxillary central incisors, which were trapped behind the lower anterior teeth.

In discussing treatment alternatives, the orthodontists focused on three risk/benefit considerations:

» Fixed appliance treatment: this was disregarded as the patient and parents felt that this would worsen the aesthetic appearance and potentially affect the patient’s self-esteem.
» Conventional removable appliance: this was disregarded as the patients and parents were concerned about the potential adverse effect on speech due to the palatal coverage.
» Invisalign^®^ appliance: the use of clear aligners would meet the demand for aesthetic treatment among both children and parents. Absence of attachments and a 5-days-change protocol were adopted to achieve treatment goals with the less burden of care.

The virtual setup (ClinCheck^®^) can display a three-dimensional image with a prediction of the final position of teeth; based on our experience, the ClinCheck^®^ itself is unlikely to have any influence of the duration of therapy, although it is potentially a very useful communication tool when obtaining consent.

The authors agree with patients and caregivers to treat this malocclusion and balance occlusal contacts with an aligner-based approach; the device was able to accomplish AC correction and intra-arch tooth alignment simultaneously. 

## ASSESSMENT

### Case 1

**Figure 1:**
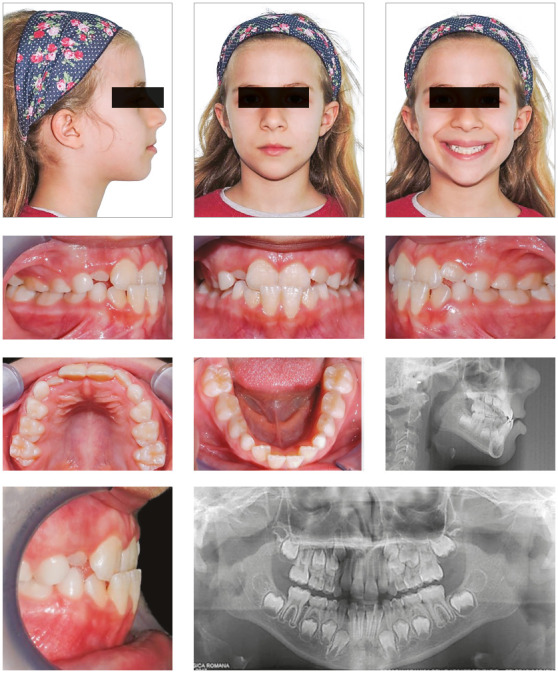
Pre-treatment intraoral and extraoral photographs, and radiographic examinations.

An 8-years-old female presented with an AC from lateral to lateral with a 1-mm negative overjet. The parents reported the absence of familiarity for Class III malocclusion. Clinical examination revealed a forward shift of the mandible due to dental interferences. Skeletal analysis: lateral cephalogram, taken in maximum intercuspation with the mandible in its displaced position, revealed a skeletal Class I (Fig 1). The Wits appraisal depicted the underlying displacement of the mandible into a tendency towards Class III. Dental analysis: The patient showed a Class I bilateral molar relationship; the AC involved both upper and lower central incisors (Fig 1). Lateral cephalogram revealed a slightly palatal inclination of upper incisors, and a labial inclination of mandibular incisors.

Soft tissue analysis: the patient’s soft tissue profile was slightly concave because of a small reduction of upper lip and a small protrusion of lower lip (Fig 1). The malocclusion was attributed to an altered eruption pattern of the permanent incisors, possibly resulting from dental crowding.

### Case 2

**Figure 2:**
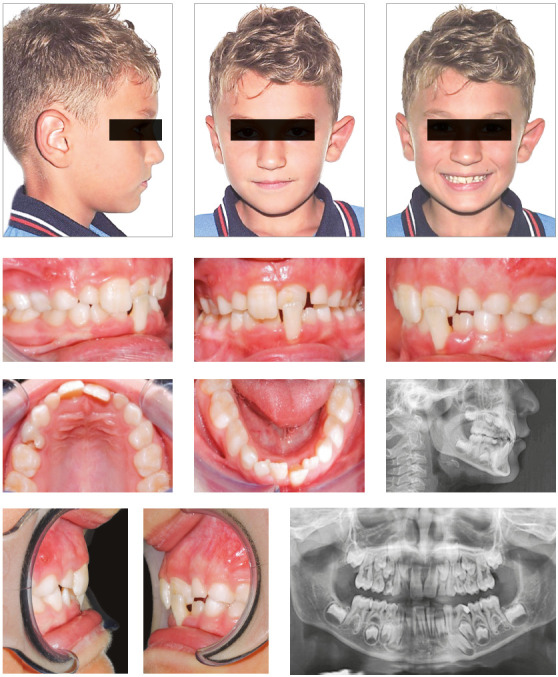
Pre-treatment intraoral and extraoral photographs, and radiographic examinations.

An 8-years-old male presented with an AC from lateral to lateral with a 1-mm negative overjet. The parents reported the absence of familiarity for Class III malocclusion. Clinical examination showed a lateral shift of the mandible due to dental interferences. Skeletal analysis: the lateral cephalogram, taken in maximum intercuspation with the mandible in its displaced position, showed a correct relationship between maxillary jaws (Fig 2).

Dental analysis: The patient showed a Class I bilateral molar relationship, and the AC involved both maxillary and mandibular central incisors (Fig 2). Lateral cephalogram revealed a slightly palatal inclination of the upper incisors, and a severe labial inclination of the left mandibular central incisor.

Soft tissue analysis: the patient’s soft tissue profile was slightly concave due to a small reduction of the upper lip and a small protrusion of the lower lip (Fig 2). The malocclusion was found to be caused by an altered eruption pattern of the permanent incisors, possibly due to dental crowding.

## TREATMENT

### Treatment objectives

Treatment goals were:

» Andrew’s third key: correct inclination of maxillary and mandibular teeth; absence of traumatic contacts.^12^
» Occlusal balance: balancing occlusal contacts to prevent functional shifts of the mandible.^12^

### Treatment plan

Diagnosis and treatment planning determine the success of AC therapy:

» Adequate space in the arch to reposition the tooth: a concomitant maxillary arch deficiency may still justify the use of rapid maxillary expansion appliances to increase available space for maxillary incisors.^13^
» Usually, aligners’ thickness provides overbite control during treatment to allow for the AC to be corrected: if the amount of vertical overbite is less than 2/3, the use of additional bite ramps should be advisable.^14^
» Occlusal relationships: to differentiate dental from skeletal crossbite, clinicians must guide the mandible into a centric relation and evaluate any change in the molar and incisor relationship from centric occlusion to maximum intercuspation, as well as estimate the relative size of the mandible compared with the maxilla.
» Castroflorio et al.^15^ recommended the use of Power Ridges (Align Technology, Amsterdam, The Netherlands) to optimize torque control. In cases with AC involving lateral incisors, the use of attachments may prevent dangerous tip movements in cases when the anterior teeth are moved prior to permanent canine eruption as the roots of the lateral incisors can be displaced into the eruption path of the canine, with the resultant risk of root resorption of the lateral incisor.

AC should be slightly overcorrected to settle the incisors into the proper relationship.

### Treatment progress

#### Case 1

Maxillary and mandibular polyvinyl siloxane impressions were taken and sent to Invisalign^®^. A virtual planning of tooth movement in three dimensions was performed through ClinCheck^®^ software (Align Technology, San Jose, CA, USA). The patient was instructed to wear each aligner 22 hours per day, even in school-time and social/sport activities. Twenty-eight aligners were scheduled, and a 5-day-change protocol was adopted.

#### Case 2

Fourteen aligners were scheduled, and a 5-day-change protocol was adopted. The overall treatment lasted 2.3 months. The child was motivated to maintain good oral hygiene. No discomfort or speech impairment were noticed by the parents.

## RESULTS

### Case 1

The overall treatment lasted 4.6 months. The child was motivated to maintain good oral hygiene. No discomfort or speech impairment were noticed by the parents.

**Figure 3:**
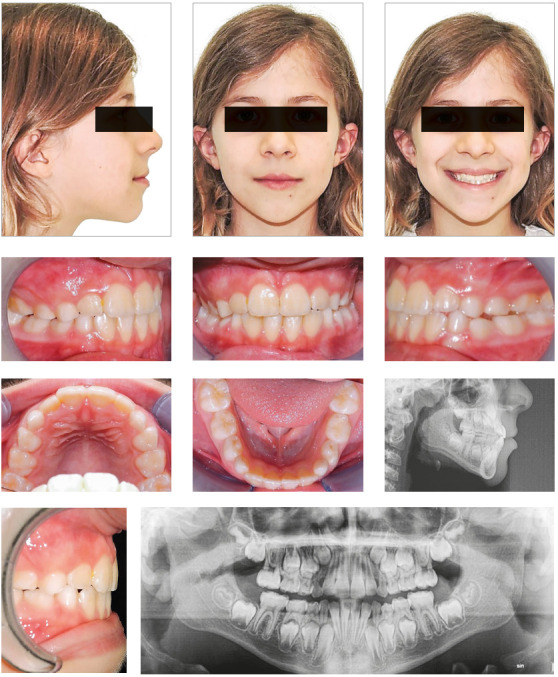
Post-treatment intraoral and extraoral photographs, and radiographic examinations

At the end of the treatment, overjet and overbite were corrected. The small skeletal improvements observed (see Wits value, Table 1) may have resulted from the elimination of the mandibular shift and change in the incisor inclination, with subsequent remodeling of the overlying alveolar bone (Table 1).The inclination of upper and lower incisors was properly settled (Fig 3). Once proper overbite was achieved, the last aligners were worn night-time for 3 months after treatment, as vacuum formed retainers.

**Table 1:** Cephalometric values.

	Patient 1		Patient 2		Normal	
VALUES	Pre-treatment	Post-treatment	Pre-treatment	Post-treatment	Mean	SD
SNA (degrees)	85	85	81	81	82	2
SNB (degrees)	82	82	78	78	80	2
ANB (degrees)	3	3	3	3	3	2
FMA (degrees)	30	28	22	22	25	3
U1-SN (degrees)	98	110	101	105	103	5
IMPA (degrees)	93	87	106	90	88	-
Wits (mm)	-4	-2	-0.5	1	2	2

### Case 2

**Figure 4:**
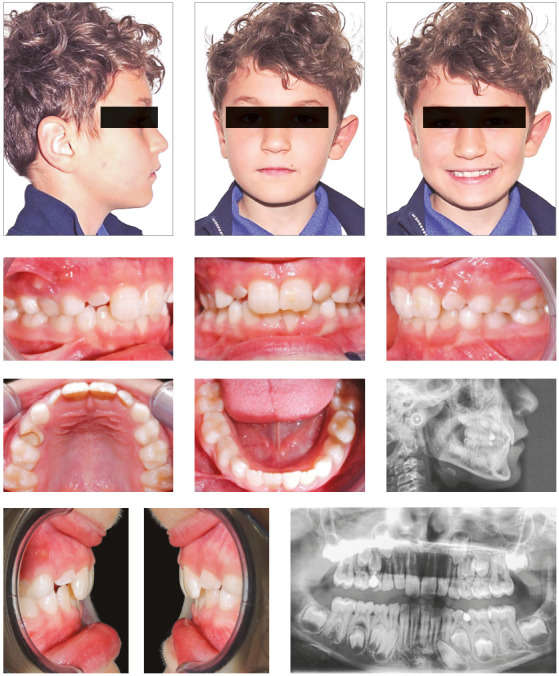
Post-treatment intraoral and extraoral photographs, and radiographic examinations.

At the end of the treatment, correct overjet and overbite were established. Moreover, the patient maintained harmonious relationships between the maxilla and the mandible (Table 1). The inclination of maxillary and mandibular incisors was properly settled (Fig 4). Once proper overbite was achieved, the last aligners were worn night-time for 3 months after treatment, as vacuum formed retainers.

## DISCUSSION

The purpose of this article was to highlight two cases of AC successfully corrected after therapy with clear aligners.

Due to the perceived shortcomings of alternative approaches, the use of clear aligners for correcting AC in mixed dentition should be considered as a comfortable and well tolerated appliance for young patients. This new technique allows young patients to participate in all their school and social activities without any aesthetic limitation. In fact, a removable device allows optimal oral hygiene, together with rigorous oral care. The use of clear aligners prevents the deterioration of periodontal status, the dental decalcifications during orthodontic treatment, and speech impairment due to the bulkiness of the removable appliance.^5^ ^,^ ^16^

Referring to the treated cases, the duration of therapy (below 5 months) was in line with conventional approaches. Li et al.^16^ showed that the amount of activation force imparted by the aligner slowly decreases and plateaued within 5 days; therefore, the aligner change protocol was optimized, stressing out that a prolonged treatment may lead to a loss of compliance, especially in young patients.

The effectiveness and efficiency of this treatment lie in its ability to achieve dental torque movements with precision.^18^ Furthermore, the occlusal vertical dimension is increased by aligners’ thickness, which prevents contacts and provides an adequate vertical clearance for a feasible crossbite correction.^19^ To avoid the use of an additional retention appliance, the final aligner can be used for three months after the end of the treatment to retain the corrected tooth positions.^20^

Some limitations of this report should also be considered. Although the results were encouraging, there is a need for evidence to draw guidelines for clinical practice and compare the perceptions of the patient’s pain and discomfort for the correction of AC with clear aligner, FA, and RA treatments.

The cost of the aligners cannot be averaged as it is fairly dependent on the company and the number of cases treated by the orthodontist. According to the prices reported by Wiedel et al,^5^ the material cost of clear aligners (above €700) is conceivably more than FA (€32) and RA therapy (€227). The cost difference is reduced if the final aligner is also used as a retainer, possibly compensating the necessity of Hawley and vacuum formed appliances.^5^

Another drawback is that a nearly full-time wear of the aligners is required to achieve an effective and efficient resolution of this malocclusion. Since clear aligners are removable devices, the orthodontic correction is entirely based on patient’s compliance.

## CONCLUSIONS

It is important to highlight the importance of clear aligners as an alternative to correct AC in mixed dentition. Notably, this technique may be easily accepted by patients who feel distressed by a fixed orthodontic treatment. Unlike removable appliances in the maxillary arch, clear aligners blend seamlessly with crown anatomy, thus avoiding the unsightly discomfort of the palatal coverage.

Short treatment time and comfortable orthodontic treatment receive positive feedback from parents and caregivers, who seek a rapid improvement in their children’ aesthetics and function.
